# Beneficial Role of Coffee and Caffeine in Neurodegenerative Diseases: A Minireview

**DOI:** 10.3934/publichealth.2016.2.407

**Published:** 2016-06-20

**Authors:** Yenisetti SC, Muralidhara

**Affiliations:** 1 Drosophila Neurobiology laboratory, Department of Zoology, Nagaland University (Central), Lumami, 798627, Nagaland, India; 2 Department of Biochemistry & Nutrition, CSIR-CFTRI , Mysore, 570020

**Keywords:** Coffee, caffeine, Neuroproective effects, animal/cell, models

## Abstract

Coffee is among the most widespread and healthiest beverages in the world. Coffee typically contains more caffeine than most other beverages, and is widely and frequently consumed. Thus, it contributes significantly to the overall caffeine consumption within the general population, particularly in adults. Controversies regarding its benefits and risks still exist as reliable evidence is becoming available supporting its health-promoting potential. Several lines of evidence have highlighted the beneficial effects towards several disease conditions including Type II diabetes, hepatitis C virus, hepatocellular carcinoma, nonalcoholic fatty liver disease and neurodegenerative disorders such as Alzheimer's disease (AD), Parkinson's disease (PD) and Amyotrophic Lateral Sclerosis (ALS). The health-promoting properties of coffee are largely attributed to its rich phytochemistry, including caffeine, chlorogenic acid, caffeic acid, and hydroxy hydroquinone. In this minireview, an attempt has been made to discuss the various evidences which are mainly derived from animal and cell models. Various mechanisms chiefly responsible for the beneficial effects of caffeine have also been briefly outlined. A short note on the undesirable effects of excessive coffee intakes is also presented.

## 1. Introduction

Coffee is among the most popular and healthiest beverages in the world [1]. Being a rich source of several biologically active natural metabolites, it possesses a variety of therapeutic effects and functional properties. Coffee has been considered as a drink which has multiple beneficial effects on human health such as cardioprotective, neuroprotective, hepatoprotective, nephroprotective, etc. Nevertheless, heavy coffee consumption may be related to some unpleasant symptoms, mainly anxiety, headache, increased blood pressure, nausea, and restlessness. During the past two decades, several lines of evidence have shown various modulatory effects of coffee (caffeine) in experimentally induced neurodegenerative conditions especially in animal models of neurodegenerative diseases (NDD). In addition, phytochemical studies showed that caffeine is the main responsible constituent for antidepressant effects of coffee through multiple molecular mechanisms.

## 2. Coffee consumption in humans

Coffee is the leading worldwide beverage after water and its trade exceeds US $10 billion worldwide. Controversies regarding its benefits and risks still exist as reliable evidence is becoming available supporting its health promoting potential. The health-promoting properties of coffee are often attributed to its rich phytochemistry, including caffeine, chlorogenic acid, caffeic acid, hydroxyhydroquinone etc. Data from animal studies, epidemiological findings and meta-analyses regarding coffee consumption have revealed its inverse correlation with that of diabetes mellitus, various cancers and NDD.

Caffeine is the most widely consumed psychoactive substance in the world. As a component of tea, coffee, and soft drinks, caffeine is the most commonly ingested methylxanthine. Caffeine consumption per capita in the United Kingdom, Sweden, and Finland is estimated to be between 100 and 400 mg per person per day, with peak consumption, where caffeine intake comes predominantly from tea and coffee, respectively. In humans, peak plasma caffeine is reached between 15 and 120 minutes after oral ingestion at doses of 5 to 8 mg/kg. The caffeine half life for these corresponding doses ranged from 0.7 to 1.2 h in rodents, 3 to 5 h in monkeys and 2.5 to 4.5 h in humans.

Over the last several decades, caffeine consumption has significantly increased. Coffee continues to be the primary source of caffeine, followed by soft drinks and tea for the average consumer in the United States [2]. Besides beverages, caffeine is now being added to food products such as potato chips, chocolates, and bottled water, confirming its growing popularity. Current estimates suggest 85% of the US population consume at least one caffeinated beverage daily, mainly sought out for its properties to improve mental alertness, concentration, and fatigue [2],[3]. While there are no specific recommendations for caffeine intakes in the U.S, the FDA has suggested that for healthy adults, caffeine intake up to 400 mg/day (around 5 cups of coffee) is not associated with adverse health effects [4].

Several epidemiological findings have suggested that coffee consumption is associated with a decrease in all-cause mortality. Multiple studies have highlighted the beneficial effects for several disease conditions such as type II diabetes mellitus, hepatitis C virus, hepatocellular carcinoma, nonalcoholic fatty liver disease and neurodegenerative disorders [2],[5]–[13]. Life-long coffee (caffeine) consumption has been associated with prevention of cognitive decline, and reduced risk of developing stroke and NDD [14]. More importantly, caffeine is shown to have several positive effects on brain function. It has been shown to increase alertness and well-being, help concentration, improve mood and limit depression. Caffeine may disturb sleep, and raise anxiety but only in sensitive individuals. Caffeine does not seem to lead to dependence, although a minority of people experience withdrawal symptoms. Caffeine is also known to potentiate the effect of regular analgesic drugs in headache and migraine.

The aim of the present review is to highlight the current understanding of the beneficial properties of coffee/caffeine and its specific role in alleviating neurodegenerative conditions. Efforts have been made to document the recent findings with specific reference to the three major neurodegenerative disorders viz., Parkinson's disease (PD), Alzheimer's disease (AD), and amyotrophic lateral sclerosis (ALS). Further, a short note on adverse impacts of coffee consumption has also been presented.

## 3. Neuromodulatory role in neurodegenerative diseases

Several epidemiological studies have associated coffee consumption with an inverse risk of developing ND. Chronic caffeine consumption has been inversely associated with the risk of developing dementia and major NDD such as AD, PD and ALS [15]–[18]. In the following sections, evidence in experimental models of NDD has been critically examined. More emphasis is laid on caffeine since a large body of experimental data is derived from studies which involve caffeine.

Caffeine has multiple targets in the brain such as adenosine, ryanodine, and γ-aminobutyric acid receptors and cyclic nucleotide phosphodiesterase isoenzymes [19]. Its action on adenosine A2a receptors may explain the psychomotor stimulant effect, mediated by dopaminergic mechanisms. Coffee contains numerous components that may also contribute significantly towards its neuroprotective effects. One of these compounds is eicosanoyl-5-hydroxytryptamide (EHT), which is demonstrated to ameliorate the phenotype associated with decreased protein aggregation and phosphorylation, improves neuronal integrity, and reduces neuroinflammation in transgenic mice [20],[21]. Chlorogenic acid, a major polyphenol contained in coffee, is known to inhibit several dopaminergic /α-synuclein-related phenomena, including the oxidation of dopamine, the interaction of oxidized dopamine with α-synuclein, and the oligomerization of α-synuclein [22]. Interestingly, caffeine is also shown to improve the sensitivity of ryanodine channels to calcium ions, and high calcium ion concentrations are harmful to motor neurons in persons with ALS [23].

### 3.1. Protective effects of Coffee, caffeine in AD and ALS

Epidemiologic studies have suggested that caffeine/coffee could be an effective therapeutic against Alzheimer's disease (AD). Studies utilising a transgenic mouse model examined if caffeine and coffee exert beneficial actions to protect against or reverse AD-like cognitive impairment and AD pathology. AD mice administered caffeine in drinking water from young adulthood into older age showed improvement in memory impairment and lower brain levels of the amyloid-beta (Aβ) protein. Moreover, “aged” cognitively-impaired AD mice exhibited memory restoration and lower brain levels of Aβ- protein following only 1–2 months of caffeine treatment [24],[25]. Previously, acute doses of caffeine were also reported rapidly to reduce the Aβ levels in both brain interstitial fluid and plasma without affecting Aβ elimination in young adult as well as aged AD transgenic mice. Further, in aged AD mice, long-term oral caffeine treatment provided sustained reductions in plasma Aβ levels and concomitantly reduced both soluble and Aβ level deposits in the hippocampus and cortex. The neuroprotective effects of caffeine were also evidenced in both 1-methyl-4-phenyl-1,2,3,6-tetrahydropyridine (MPTP) and 6-hydroxydopamine (6-OHD) models [26]. A recent study has demonstrated that caffeine and A2A R inactivation decreases Machado-Joseph disease (MJD)-associated striatal pathology [27]. MJD is a neurodegenerative disorder associated with an abnormal CAG expansion, which translates into an expanded polyglutamine tract.

Caffeine has been shown to play a role in improving memory performance and exerts protective effects against AD by increasing the expression or activity of Na^+^-K^+^-ATPase [28]. In a recent study, Han et al [29] found that the long-term consumption of caffeine increased CSF production with a concomitant increase in the expression of Na^+^-K^+^-ATPase and increased cerebral blood flow. Long-term caffeine consumption could exert protective effects against AD at least in part by facilitating CSF production [30]. Caffeine and SCH58261, modulators of adenosinergic receptors, were able to reverse age-associated memory impairment and also normalized Na^+^-K^+^-ATPase activity [31].These findings clearly suggest that caffeine may play a role in ALS by normalizing Na^+^-K^+^-ATPase activity.

Several findings suggest that caffeine may play a significant modulatory role in ALS via various mechanisms. Glutamate, an excitatory neurotransmitter has been proposed to play a significant role in ALS. Chronic neuroinflammation is associated with an increase in extracellular levels of glutamate and drugs that limit the effects of glutamate at neuronal receptors have been shown to indirectly reduce the neuroinflammatory response of microglia cells. Interestingly, caffeine attenuated the number of activated microglia within the hippocampus of animals with LPS-induced and age-related inflammation [32]. Caffeine attenuates excitatory amino acid transporter type 3 (EAAT-3) activity and this reducing effect of caffeine was shown to be mediated by protein kinase C (PKC) and phosphatidylinositol 3-kinase (PI3K) in Xenopus oocytes [33]. In another study, caffeine decreased the reserpine-induced increase in extracellular glutamate in the striatum of rats with an alteration of vesicular monoamine transporter (VMAT2) function [34]. Maternal caffeine intake during gestation causes downregulation of A1and metabotropic glutamate receptors in the brain of both rat mothers and fetuses [35]. Caffeine also affects receptors in the heart, causing a decrease in metabotropic glutamate receptors (mGluRs) from both maternal and fetal hearts [36], suggesting an in vivo cross-talk mechanism between adenosine and glutamate receptors in peripheral tissues.

*Evidence in cell models:* The molecular events underlying the protective effect of caffeine have been investigated in various cell models. Caffeine inhibited ultraviolet (UV)-induced phosphorylation of p38 MAPK in A2058 melanoma cells [37] and also inhibited UV-induced activation of SEK, an upstream MAPK kinase of JNK, resulting in suppression of both K^+^-channel-involved and DNA damage-induced p53 activation [38]. In osteosarcoma cells lines, caffeine inhibited proliferation and suppressed nuclear factor κB (NF-κB), AKT, and ERK activities [39]. Caffeine treatment stimulated cAMP-dependent protein kinase A (PKA) and phospho-cyclic AMP response element binding protein (phospho-CREB) and decreased phospho-JNK and phospho-ERK expression in the striatum of AD transgenic mice [40]. Caffeine treatment repressed extracellular signal-regulated kinase (ERK)-mediated c-Fos phosphorylation but evoked p38 MAPK-mediated c-Jun phosphorylation [41]. Caffeine also attenuated the lipopolysaccharide (LPS)-induced phosphorylation of ERK in microglial cells [42].

### 3.2. Protective effects in PD models: Effect of EHT

Consumption of coffee is reported to be associated with reduced risk of Parkinson's disease (PD), an effect that has been attributed to caffeine. Evidence suggest that EHT, one of the compounds present in coffee is known to be chiefly responsible for its protective effect against PD. It ameliorates the phenotype in transgenic mice and significantly decreases protein aggregation and phosphorylation, improved neuronal integrity and reduced neuroinflammation. In a recent study [43] the modulatory effect of EHT was investigated in an MPTP model of PD. Mice fed a diet containing EHT for four weeks exhibited dose-dependent preservation of nigral dopaminergic neurons following MPTP challenge compared to animals given control feed. Reductions in striatal dopamine and tyrosine hydroxylase content were also less pronounced with EHT treatment. EHT not only markedly attenuated, the neuroinflammatory response, but also reduced indices of oxidative stress and JNK activation. Further this study also demonstrated that EHT had a direct anti-inflammatory effect in cultured primary microglia and astrocytes, as evidenced by the repression of lipopolysaccharide-induced NFκB activation, iNOS induction, and nitric oxide production. Further, EHT also exhibited robust antioxidant activity in vitro. Additionally, EHT ameliorated MPP(+)-induced demethylation of phosphoprotein phosphatase 2A (PP2A), and cytotoxicity in SH-SY5Y cells.

The pathophysiology of PD is largely due to the nigrostriatal DA system, with a decrease in the activity, synthesis, and mRNA levels of TH in the striatum of PD and experimental animal models [44],[45]. Tyrosine hydroxylase (TH) is the rate-limiting enzyme in the biosynthesis of dopamine and other catecholamines. Chronic caffeine intake prevented the degeneration of DA cell bodies in the SN of rats following chronic intracerebroventricular infusion of MPTP [46]. Earlier studies have shown that caffeine stimulates Ca^2+^ entry through store-operated channels to activate TH in bovine chromaffin cells [47]. Animals that received caffeine for nine consecutive days at doses of 20, 40, and 80 mg/kg of body weight displayed increased TH mRNA levels in the SN and the ventral tegmental area [48]. Further, in an experimental model of PD induced by pesticides, Paraquat and Maneb, caffeine (20 mg/kg) significantly reduced the TH immunoreactivity and loss of dopaminergic neurons [49].

## 4. Mechanisms underlying the neuromodulatory effects of Coffee( caffeine)

Various mechanism/s have been explained to be largely contributing towards the beneficial effects of caffeine and the evidences are derived from studies in both cell models and animal models. The major mechanisms comprise of the specific effects of caffeine on neurotrophic factors, Poly (ADP-Ribose) Polymerases, Vascular Endothelial Growth Factor, inflammatory processes and anti-oxidative defenses. These have been briefly discussed below.

### 4.1. Modulatory effect of Caffeine on neurotrophic factors

Several earlier studies have shown that acute treatment with caffeine improves recognition memory, and caffeine prevents age-associated recognition memory decline and changes BDNF and TrkB content in mice [50],[51]. The effects of caffeine were significant and prevented the weight-gain associated with a high-fat diet and cognitive impairment. The experimental evidence clearly demonstrated that chronic caffeine treatment prevented the impairment of long-term memory as measured by performance in the radial arm water maze task and normalized late phase long-term potentiation (LTP) in area CA1 of the hippocampi of sleep-deprived anaesthetized rats. Further, Caffeine treatment substantially reduced age-related impairments in memory in an inhibitory avoidance paradigm and concomitantly elevated the BDNF levels in the hippocampus [52] or BDNF mRNA and protein levels in the carotid body and nucleus tractus solitaries of female rats [53]. Another study showed that caffeine prevented stress-induced LTP impairment in rats. Western blot analysis showed a reduction of the basal levels of the phosphorylated calcium calmodulin kinase II (P-CaMKII), total CaMKII, and BDNF in area CA1 of stressed rats [54].

Neurotrophic factors (NTFs) such as NGF, BDNF, NTF-3, and GDNF are keys to surviving various neuronal insults, and they promote neuronal regeneration following injury. Chronic caffeine treatment was shown to significantly reverse memory impairment and the expression of BDNF and TrkB in a transgenic mouse model of neurodegenerative disease with a dose-response effect [29]. Likewise, caffeine also restored a decrease in hippocampal BDNF seen in high-fat-fed animals [55].

### 4.2. Caffeine and Poly(ADP-Ribose) Polymerases(PARPs)

Caffeine metabolites are inhibitors of PARPs, which are involved in a wide range of molecular and cellular processes, including maintenance of genome stability, regulation of chromatin structure and transcription, cell proliferation and apoptosis [56]. The major caffeine metabolite 1,7-dimethylxanthine has significant PARP-1-inhibiting activity in cultured epithelial and endothelial cells at physiological concentrations [57]. Caffeine at a concentration of 100 mM inhibited PARP-1 synthesis in permeable cells [58].

### 4.3. Caffeine and Vascular Endothelial Growth Factor

Several studies suggest that caffeine may modulate the expression of Vascular endothelial growth factor (VEGF), a key mediator of angiogenesis. Angiogenesis is a complex process that involves coordinated steps of endothelial cell activation, proliferation, migration, tube formation, and capillary sprouting. Previous studies have shown that adenosine up-regulates VEGF expression in cultured myocardial vascular smooth muscle cells and human glioblastoma cell lines [59],[60]. Adenosine is known to interact with four subtypes of G- protein-coupled receptors, termed A1, A2A, A2B, and A3. A2AR activation induces VEGF in human retinal endothelial cells [61]. Caffeine inhibits the adenosine-induced accumulation of hypoxia-inducible factor 1α, VEGF, and interleukin-8 expression in hypoxic human colon cancer cells [62]. Pretreatment of the human fetal kidney cell line with caffeine resulted in complete inhibition of hypoxia-induced VEGF gene expression [63].

### 4.4. Effects on inflammatory processes

Caffeine substantially suppressed the LPS-induced pro-inflammatory mediators PGE2 and tumor-necrosis factor α (TNF-α) in BV2 microglial cells [42]. Caffeine exerts its effects on macrophages by altering the cAMP level and PG synthesis [64]. Caffeine is often used in combination with other analgesics, which augments their effect. Both paracetamol and caffeine dose-dependently inhibited microglial PGE2 synthesis. In combination with acetylsalicylic acid, both the substances augmented the inhibitory effect of acetylsalicylic acid on LPS-induced PGE2-synthesis. While paracetamol inhibited only COX enzyme activity, caffeine inhibited COX-2 protein synthesis as well [65].

### 4.5. Coffee and oxidative stress mechanisms

In an animal model of ALS, coffee was found to elevate the antioxidant enzyme capacity significantly in the brains of male G39A mice which showed improved motor performance [66]. When applied to human neuronal SH-SY5Y cells, the major components of energy drinks (caffeine, taurine, and guarana) induced a concentration-dependent non-enzymatic antioxidant potential, decreased the basal levels of free radical generation, and reduced the activity of enzymes such as superoxide dismutase (SOD) and catalase (CAT) especially when combined together [67]. Caffeine in combination with indomethacin (an inhibitor of PG synthesis), exhibited a higher effect as evidenced by increased cellular viability, reduced superoxide anion production and DNA fragmentation [64].

In recent studies, coffee chlorogenic acid, its derivatives and certain caffeine metabolites were shown to reduce significantly some of the free radical damage sustained to DNA [68]. Caffeine protects human skin fibroblasts from acute ROS-induced necrosis [69]. Additionally, methylxanthine caffeine inhibits the DNA damage response in vitro and in vivo, regulates both cell proliferation and apoptosis after DNA damage, inhibited ROS levels and reduced atherogenesis in ApoE-/-mice [70]. Pretreatment with caffeinated coffee, decaffeinated coffee or chlorogenic acid also inhibited the H2O2-induced downregulation of the antiapoptotic proteins- Bcl-2 and Bcl-X(L) while blocking proapoptotic cleavage of caspase-3 and poly (ADP-ribose) polymerase [71]. Treatment with caffeine (or a selective A2A receptor antagonist) significantly normalized the levels of ROS oxygen and reactive nitrogen species that are usually increased in the brains of aged rats [31].

## 5. Effects of coffee on cognition and psychomotor behavior

Moderate (3–5 cups a day) coffee consumption in humans is associated with a significant decrease in the risk of developing certain chronic diseases. However, the ability of coffee supplementation to improve cognitive function in aged individuals and the effect of the individual components in coffee (e.g., Caffeine), has not been thoroughly evaluated. A recent study investigated the effect of coffee on cognition and behaviour. They fed aged rats one of five coffee-supplemented diets (0, 0.165, 0.275, 0.55, and 0.825%) for eight weeks and monitored motor and cognitive behavior [72]. Aged rats supplemented with a 0.55% coffee diet (equivalent to 10 cups of coffee) performed better in psychomotor testing (rotarod) and in a working memory task (Morris water maze) compared to aged rats fed a control diet. The 0.165% coffee-supplemented group (3 cups) showed some improvement in reference memory performance in the Morris water maze. In a subsequent study, the effects of caffeine alone did not account for the performance improvements, showing that the neuroprotective benefits of coffee are not due to caffeine alone, but rather due to other bioactive compounds in coffee.

## 6. Undesirable Side Effects of coffee/caffeine

Several reports indicate that excessive intake of caffeine is associated with anxiety, headaches, nausea, and restlessness [3],[73]. Side effects (i.e., headache, fatigue, drowsiness) are reported to be experienced when caffeine intake is stopped suddenly, although symptoms are mild and temporary. Some but not all studies have shown an increased risk of hypertension and cardiovascular disease [3],[74]. Moderate caffeine intake (less than 400 mg/day for healthy adults) does not adversely affect cardiovascular health. Scientific data do not support adverse effects of moderate caffeine consumption below 300 mg/day on reproductive health or pregnancy outcomes [75],[76].

Tolerance to the psychostimulant and cardiovascular effects of caffeine usually develops within a couple of days. High-dose caffeine intake has been reported to elicit symptoms of nervousness, agitation, anxiety and insomnia, a syndrome called caffeinism. The majority of patients suffering from caffeinism develop a variety of nervous, gastrointestinal, or cardiac symptoms after consumption of differing quantities of caffeine, usually more than 250 mg. Acute states of confusion also have been associated with very high levels of caffeine intake, more than 1000 mg per day. Anxiety and somatic abnormalities have been observed in regular coffee drinkers even after absorption of small quantities of caffeine (< 250 mg), but these people most likely were very sensitive to caffeine effects. Caffeinism also has been associated with delirium, psychoses, and anorexia nervosa. Finally, several cases of death have been reported following intravenous and oral absorption of an excessive amount of caffeine (5–10 g). Symptoms observed in caffeine poisoning are agitation, anxiety, convulsions, tachycardia, and coma, with death by pulmonary edema, ventricular fibrillation, and cardiopulmonary arrest.

## 7. Summary and Future perspectives

Since coffee (Caffeine) is a widely consumed food constituent and can exert a variety of physiological effects, it has generated great research interest. Despite the longstanding consumption of caffeine-containing beverages in the diet, there is a lack of comprehensive and current population-based data on caffeine intakes. The introduction of functional beverages such as energy drinks, energy shots, as well as a variety of speciality coffees, emphasises the need to understand and characterise the beneficial effects of coffee on the brain physiology and neurodegenerative disorders. This assumes great relevance since epidemiological evidence indicates an inverse correlation between coffee (caffeine) consumption and NDD risk. Comprehensive efforts need to be directed towards understanding the potential interaction between various protective compounds present in coffee and more randomised trials are required to assess the effects of caffeine in the prevention of NDD such as AD and PD.
